# Relationship between Arterial Calcifications on Mammograms and Cardiovascular Events: A Twenty-Three Year Follow-Up Retrospective Cohort Study

**DOI:** 10.3390/biomedicines10123227

**Published:** 2022-12-12

**Authors:** Natalia González Galiano, Noemi Eiro, Arancha Martín, Oscar Fernández-Guinea, Covadonga del Blanco Martínez, Francisco J. Vizoso

**Affiliations:** 1 Department of Internal Medicine, Fundación Hospital de Jove, Av. Eduardo Castro, 161, 33290 Gijón, Spain; 2 Research Unit, Fundación Hospital de Jove, Av. Eduardo Castro, 161, 33290 Gijón, Spain; 3 Department of Emergency, Hospital Universitario de Cabueñes, Los Prados, 395, 33394 Gijón, Spain; 4 Department of Radiology, Fundación Hospital de Jove, Av. Eduardo Castro, 161, 33290 Gijón, Spain; 5 Department of Surgery, Fundación Hospital de Jove, Av. Eduardo Castro, 161, 33290 Gijón, Spain

**Keywords:** breast arterial calcifications, cardiovascular risk, mammograms, risk assessment, prevention, cost effectiveness

## Abstract

Purpose: Breast arterial calcifications (BAC) have been associated with cardiovascular diseases. We aimed to examine whether the presence of BAC could predict the development of cardiovascular events in the very long term, as evidence has suggested. Patients and Methods: We conducted a 23-year follow-up retrospective cohort study considering women specifically studied for breast cancer. After reviewing the mammograms of 1759 women, we selected 128 patients with BAC and an equal number of women without BAC. Results: Women with BAC had higher relative risk (RR) for cardiovascular events, globally 1.66 (95% CI): 1.31–2.10 vs. 0.53 (0.39–0.72), and individually for ischemic heart disease 3.25 (1.53–6.90) vs. 0.85 (0.77–0.94), hypertensive heart disease 2.85 (1.59–5.09) vs. 0.79 (0.69–0.89), valvular heart disease 2.19 (1.28–3.75) vs. 0.83 (0.73–0.94), congestive heart failure 2.06 (1.19–3.56) vs. 0.85 (0.75–0.96), peripheral vascular disease 2.8 (1.42–5.52) vs. 0.85 (0.76–0.94), atrial fibrillation 1.83 (1.09–3.08) vs. 0.86 (0.76–0.98), and lacunar infarction 2.23 (1.21–4.09) vs. 0.86 (0.77–0.96). Cox’s multivariate analysis, also considering classical risk factors, indicated that this BAC was significantly and independently associated with survival (both cardiovascular event-free and specific survival; 1.94 (1.38–2.73) and 6.6 (2.4–18.4)). Conclusions: Our data confirm the strong association of BAC on mammograms and the development cardiovascular events, but also evidence the association of BAC with cardiovascular event-free and specific survival.

## 1. Introduction

Medicine needs to optimize health resources. In this way, mammograms represent the most valid test for detecting breast cancer, with good acceptance, minimal adverse effects, and low economic costs. Moreover, mammograms can recognize other morphological findings, such as breast arterial calcifications (BAC), the presence of which could predict cardiovascular risk [1]. BAC are a common finding on mammograms, seen as parallel radiopaque structures or tubular tracts frequently involving the entire circumference of the artery and easy to detect. The prevalence of BAC is estimated to be between 8.2% and 12% among women over 50 [1].

BAC correspond to the calcification of the arterial middle layer, or Mönckeberg’s arteriosclerosis, an early histological change where vascular smooth muscle cells are similar to matrix bone, without macrophages and lipids [2,3].

As some studies have shown, there is an association between the presence of BAC and renal disease [4], stroke [5], peripheral vascular disease, low bone mass, carotid artery narrowing and, mainly, coronary artery disease [6,7,8,9,10]. Several studies have shown a strong association between the presence of BAC and cardiovascular mortality [1,11,12,13]. It has been reported that the finding of BAC on mammograms of women under 59 years could be an additional risk factor for cardiovascular diseases [14], especially in diabetic patients [13]. Cardiovascular mortality increased by a 40% in women with BAC compared to women without BAC, reaching the 90% in diabetic women [1,12]. More recent studies confirm the evidence of a relationship between the presence of BAC and coronary disease in asymptomatic women [15,16].

We previously reported an association between BAC and biochemical markers of endothelial injury (higher serum levels of triglycerides, homocysteine, hs-CRP and an elevated LDL-C/HDL-C ratio (coronary risk index > 2)) [17] and between BAC and aged-related macular degeneration [18]. However, despite all of these data concerning the relationship between BAC and the occurrence of cardiovascular risk factors and cardiovascular disease, there is little information between this radiographic finding and the subsequent development of cardiovascular events and the associated mortality. Hence, the aim of the present study was to evaluate these clinical associations in a cohort of women after a long period of time.

## 2. Methods

### 2.1. Patient Selection

We conducted a retrospective cohort study in a regional hospital. Considering that BAC were not a commonly described finding in mammographic reports many years ago, we were able to carry out the present study with such a long follow-up period thanks to our previous works about the relationship between BAC and biochemical markers of endothelial injury [17]. In this prior study, we reviewed the mammograms of 1759 women, made between June 1996 and June 2004, in a screening breast cancer program. We detected BAC in 147 women. We contacted these patients between January and March 2021. A total of 19 cases were lost in the BAC female population; 10 women refused to communicate because of the anxiety caused by the pandemic circumstances, and 9 other candidates could not be located. Finally, a cohort of 128 women with BAC in the baseline mammograms or their families were contacted and agreed to participate in the present study, and they form the main study group. Due to difficulties in following up all initially studied subjects, an equal number of women without BAC were completed and selected from the initial population (1615 remaining women without BAC). There was an exact coincidence for mean age (the women without BAC age matched to the women with BAC) and a similar distribution for baseline clinical characteristics, such as hypertension, diabetes mellitus (DM), dyslipidemia (DL), or smoking, between both women groups. We investigated the evolution of all the women in terms of the development of cardiovascular events and their associated mortality over such a long time (a mean of 23 years of follow up, range 16–26 years, from June 1996 to March 2021).

### 2.2. Data Collection

BAC were identified as breast tissue opacities affecting all of circumference or seen as parallel tubular opacities. BAC was classified as present or absent, as recommended by the American College of Radiology for breast cancer screening [19]. None of these patients showed changes in the BAC status with regard to the latest mammogram, performed either 1 or 2 years earlier. Considering the long time of the study, we evaluate the initial analog mammography for classifying women as having BAC and not. Senographe 600T and Senographe 800T (0.3 mm focal spot and 0.1 mm for magnification) and Mamoray films, screens, and cassettes (18 cm × 24 cm) were used to perform mammography. To reduce scatter radiation, a grid was used to make a vigorous breast compression. Two basic projections (mediolateral oblique and craniocaudal views), on semiautomatic to automatic exposure mode applying 27 to 30 keV were performed. Additional projections and accessory magnification images were performed when needed. The mammography image was reviewed by two expert radiologists (O.F.-G. and C.d.B.M.), independently, with extensive experience in this area. The reading was blinded for both of them; they had no knowledge about any clinical data. The concordance was greater than 95%. When there was a disagreement in their interpretation (present vs. absent), authors revised the image together to reach an agreement.

The baseline variables recovered were the age and clinical-pathological characteristics: hypertension, diabetes mellitus (DM), hypercholesterolemia (DL), smoking, cardiovascular diseases among first-degree relatives, weight, height, and body mass index. These same clinical variables were recorded from the medical health records of each woman at the end of the follow-up period. Hypertension was defined as a systolic blood pressure greater than 140 and diastolic blood pressure greater than 90 mmHg. The diagnostic criteria for DM were: random venous blood glucose greater than 200 mg/dL, associated with classic symptoms such as polyuria, polydipsia, polyphagia, weight loss, and asthenia; fasting venous blood glucose greater than 125 mg/dL; a glycosylated hemoglobin (HbA1c) greater than 6.5%, and a blood glucose level two hours after an oral glucose stress test greater than 200 mg/dL. A history of DL was considered if total blood cholesterol values were greater than 250 mg/dl and/or triglyceride values were greater than 200 mg/dl. We consider date on therapies. A history of cardiovascular risk or cardiovascular disease was considered as present if a woman was taking any medical treatment for that. Body mass index (BMI) was calculated as body weight in kilograms divided by height in squared meters (BMI = weight/height [2] (kg/m^2^). Women were considered obese if BMI calculation was equal or greater than 30 kg/m^2^. Women who smoke every day or stopped smoking less than 6 weeks before the study began were considered smokers.

The occurrence of cardiovascular diseases, such as ischemic, hypertensive, and valvular heart diseases, congestive heart failure, peripheral vascular disease (PVD), and cerebrovascular accident (ischemic, hemorrhagic, and lacunar infarction), were confirmed from the patient’s medical history and considered as events during all the follow up period. The development of atrial fibrillation was also registered and included as an endpoint in consideration of its importance in the risk of sudden cardiac death [20]. We considered the date of the initial prescription. Both the events and the causes of death were extracted manually from the medical records where they are coded by the ICD-10 nomenclature.

### 2.3. Statistical Analysis

Statistical analysis was performed by N.E. using the SPSS for Windows software, version 25 (Chicago, Illinois). The sample size was calculated from the established population of women specifically studied for early breast cancer screening. The Kolmogorov–Smirnov test was used to determine data distribution. Non-normally distributed data are expressed as median-range, whereas categorical variables are displayed as numbers and percentages. There were no missing data. Non-parametric Mann–Whitney U tests were used to determine the differences between mean values for non-normally distributed variables. Since this is a cohort study, the risk ratio was used to comparatively evaluate the development of events in each group. For survival analysis Cox’s univariate method was used. In the case of cardiovascular event-free survival analysis, the first cardiovascular event in each woman was considered as end-point, and we considered the time to event since the first mammography was performed. Cox’s regression model was used to examine interactions of different risk factors in a multivariate analysis. Only factors that achieve statistical significance in the univariate analysis were included in the multivariate analysis (Cox’s regression model). *p* ≤ 0.05 was considered as significant. The significance level was stablished at level *p* < 0.05. The PASW (Predictive Analytics Software) statistics 18 program (SPSS Inc., Chicago, IL, USA) was used for all calculations.

### 2.4. Ethical Aspects

Following the guidelines of the Ethics Committee of our institution, in accordance with the Declaration of Helsinki, informed consent was obtained from each participant. The family was contacted to sign the informed consent if the patient had cognitive impairment or had died. Accurate and complete information was gathered from interconnected electronic health records between the hospital and general primary care centers, preserving their confidentiality in accordance with institutional regulations. N.G.-G. was authorized to consult these records.

## 3. Results

### 3.1. Baseline Clinical Characteristics of the Patients and at the End of the Follow-Up Period

Table 1 shows clinical-pathological characteristics of 128 women in each group. The mean age at the baseline mammogram was 59 years (44–74) in the BAC group and 59.5 years (44–70) in the control group. Both groups displayed a similar distribution of classical cardiovascular risk factors, such as hypertension, DM, DL, obesity, and smoking.

Regarding the number of cardiovascular risk factors recorded between the two groups at the end of the 23-year follow-up period, no significant differences were found, except for hypertension (Table 1).

### 3.2. Development of Cardiovascular Events during the Follow-Up Period

**Figure 1:**
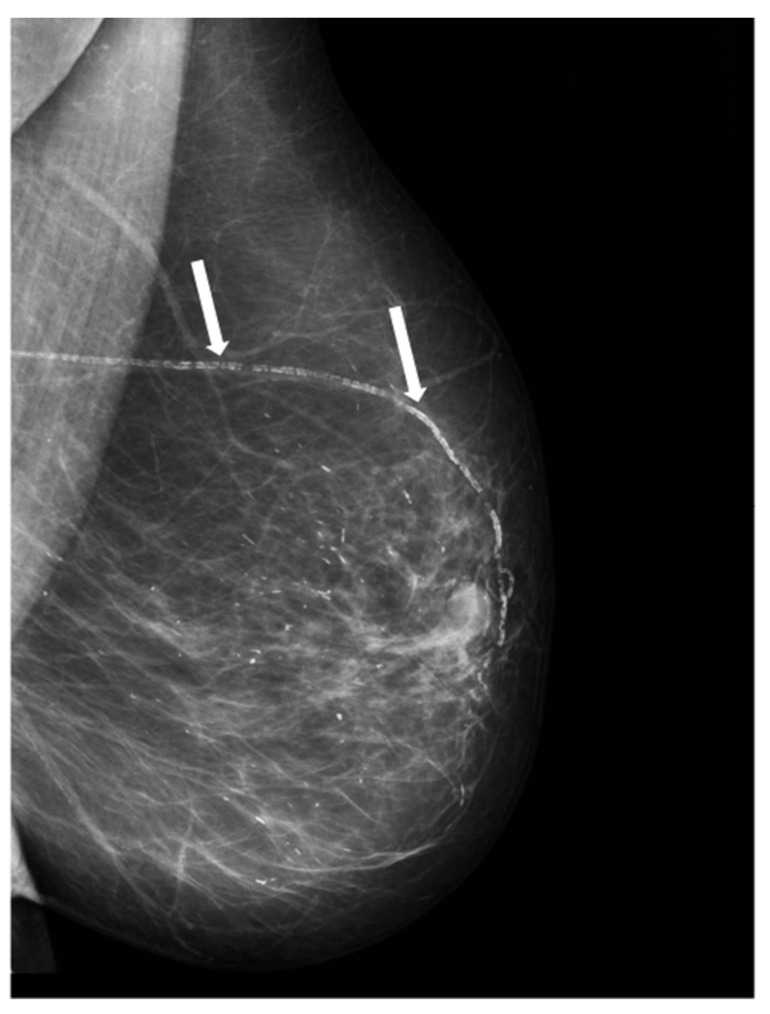
Mammogram showing breast arterial calcifications (rows).

**Figure 2:**
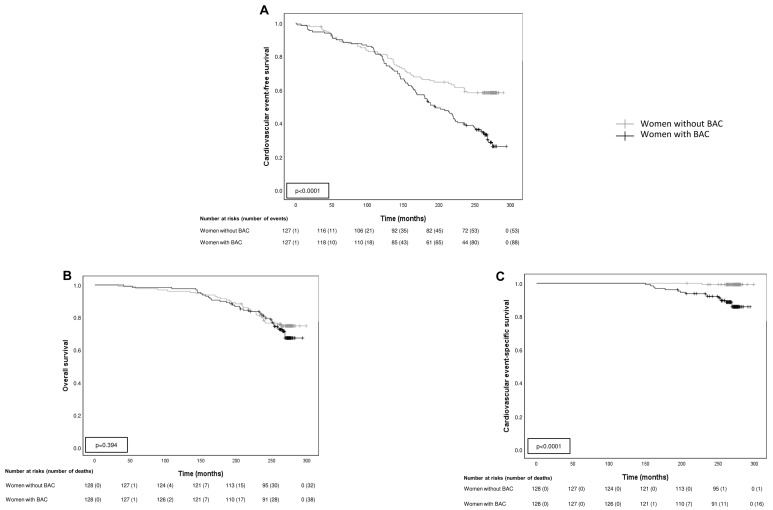
Kaplan-Meier survival curves. (**A**) Kaplan-Meier cardiovascular events-free survival curve as a function of patients with or without BAC. Kaplan-Meier survival curve as a function of patients with or without BAC. (**B**) overall survival and (**C**) cardiovascular event-specific survival.

Table 2 shows the number of cardiovascular events during the follow-up period of 23 years for each group. The identification of BAC in the baseline mammogram (Figure 1) was strongly associated with the incidence of cardiovascular events. Thus, a total of 88 (68.8%) women with BAC developed at least one of these events compared to 53 (41.4%) women in the BAC-free group. In addition, our data also shows significant differences between cardiovascular events-free survival curves calculated for both groups (*p* < 0.0001) (Figure 2A). Globally, over the follow-up period, the BAC group had a higher global incidence of cardiovascular events than women without BAC (total number: 232 vs. 102) (*p* < 0.0001) (Table 2). In the BAC group, 19 (14.8%) women had one event, 28 (21.9%) two events, 22 (17.2%) three events, and 21 (16.4%) four or five events, whereas in the BAC-free group, 21 (16.4%) women had one event, 22 (17.2%) two events, seven (5.5%) three events; and four women (3.1%) four events. In addition, as can be seen in Table 2, women with BAC had a higher relative risk (RR) of global cardiovascular events compared with women without BAC, as well as specifically for each event type. Women with BAC have a higher RR of developing ischemic heart disease, hypertensive heart disease, valvular heart disease, congestive heart failure, atrial fibrillation, PVD, and lacunar infarction, as well as higher mortality due to cardiovascular events (RR: 13.13 (1.84–238) vs. 0.61 (0.47–0.79)). However, we found no significant difference between the groups for ischemic or hemorrhagic cerebral events.

### 3.3. BAC Influence on Survival

**Figure 3:**
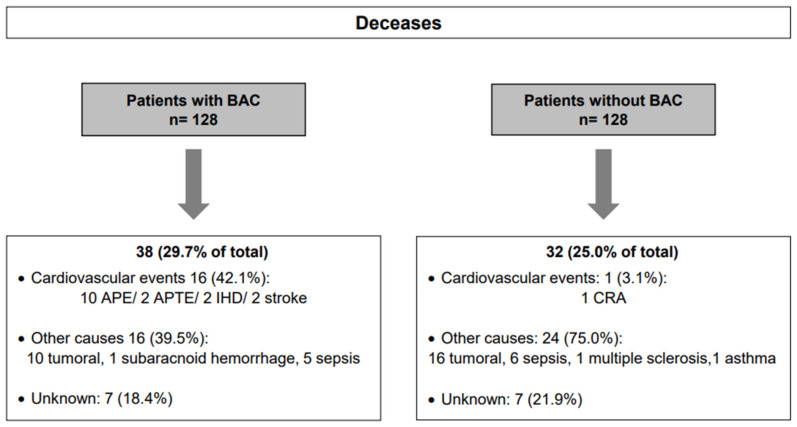
Overview of deceases and causes in patients with or without BAC. BAC: Breast arterial calcification, APE: Acute pulmonary edema; APTE: Acute pulmonary thromboembolism; IHD: Ischemic heart disease; CRA: Cardiorespiratory arrest.

During the study period, there were 38 deaths (29.7%) in the BAC group and 32 (25%) in the BAC-free group (Figure 2B-overall survival curve). Nevertheless, as illustrated in Figure 2C, there were significant differences between cardiovascular event-specific survival curves calculated for both patient groups (*p* < 0.0001). There was a higher number of deaths because of cardiovascular events in women with BAC (16 (42.1%) vs. 1 (3.1%)) (Figure 3). There were no statistically significant differences between the age of death in both groups (*p* = 0.706).

Table 3 shows Cox’s multivariate (hazard ratio) analysis of the relationship between classical cardiovascular risk factors present at the baseline study, cardiovascular event-free survival, and cardiovascular event-specific survival. The results show that DM and BAC were significantly and independently associated with both survival analysis, whereas hypertension was also significantly and independently associated with event-specific survival. This analysis also demonstrated that BAC was a significant and independent factor to predict both survival variables.

## 4. Discussion

This is one of the first studies evaluating, over such a long period of time, the impact of BAC for the development of cardiovascular events and their associated mortality in a cohort of women studied specifically for its radiological finding. Our results, showing a very strong and positive association between BAC, cardiovascular events and associated mortality, are consistent with previous studies [21,22]. Even the association between BAC and coronary artery disease was stronger compared with traditional Framinghan risk factors [23]. In accordance with this, Chadashvili et al. found out that BAC predicted a coronary artery calcium score over 11, which indicates a moderate or severe risk of developing coronary artery disease [7]. In addition, recent data from over 45-year-old women show a significant correlation between the severity of BAC and the extent of coronary artery disease (verified by coronary angiography defined by the SYNTAX classification) [24]. On the other hand, in our study, the presence of BAC at the baseline mammogram was also very positively and strongly associated with other cardiovascular events, such as valvular disease, heart failure, PVD, and cerebral lacunar infarction, as well as atrial fibrillation, compared to the control group. All these events in the group of women with BAC were not influenced by the emergence of new risk factors throughout the 23 years of follow-up, except for hypertension. In accordance with these findings, our results indicate that BAC was an independent prognostic factor to predict not only the development of cardiovascular events, but also a higher probability of derived death.

Recently, Iribarren et al. reported the results of a similar study but after a mean follow-up of 6.5 years [16]. They noticed a higher global cardiovascular events among women with BAC than those without BAC, statistically significantly (*p* ≤ 0.04) for ischemic stroke, cardiovascular death, cerebrovascular disease, cardiomyopathy, deep venous thrombosis/pulmonary embolism, peripheral arterial disease, and retinal vascular occlusion. However, while in their study there was no clear separation of survival curves between the two groups, we found significant differences (*p* < 0.0001), with a higher number of deaths from cardiovascular outcomes in women with BAC (16 (42.1%) vs. 1 (3.1%)), probably due to the longer follow-up period. On the other hand, although women without BAC had similar baseline classical risk factors compared with women with BAC, our data show that they develop less frequently hypertension at the end of the follow-up period (*p* = 0.049) compared with women with BAC. This finding could contribute to some protection against cardiovascular events. Nevertheless, further investigation might to identify other protective factors in these women without BAC.

The findings of the present study are particularly relevant considering that cardiovascular diseases are the leading cause of mortality worldwide. It is estimated that by 2030, approximately 23.3 million people will die from cardiovascular diseases. In addition, it is relevant to consider that coronary disease is the main cause of death among women, as well as this process has a worse prognosis than for men [21]. Therefore, quantifying the importance of cardiovascular diseases and their main risk factors is an essential aspect of proper planning of existing health resources, since nearly 50% of cardiovascular mortality reductions are due to the control of its major risk factors. However, the problem is that many patients ignore that they suffer from any of these disorders, such as 50% of hypercholesterolemic, a third of hypertensive, or 20% of diabetics. Therefore, the BAC detected by mammography could represent a diagnostic axis on which to focus cardiovascular disease prevention policies.

Mammography, reflecting the composition of a part of the human tissues, offers us the possibility to use this diagnostic platform to recognize another morphological finding of interest that is not usually recorded in mammographic reports, such as BAC. In addition, there are data which support a stronger association between BAC and cardiovascular mortality, compared to arterial calcification in other locations, such as aortic, splenic, internal and external iliac, detected by computed tomography [8]. Millions of women worldwide undergo a mammogram annually or biannually. Correspondingly, there is a large amount of available data in digital files from studies which could be already conducted. The tools of current computer applications and modern artificial intelligence makes easier to manage all these massive digital data. Combined with clinical data, it could help us to create different universal algorithms to identify women who may benefit from therapeutic or preventive health measures. On the other hand, there are other possibilities for using resources based on the associations previously described for BAC, such as the possibility of identifying women with low bone mass [24], predicting the course of chronic kidney disease [25], or the cardiotoxic effect of anthracycline- derived agents, trastuzumab, and radiotherapy in the treatment of breast cancer [26].

Limitations of our study are its retrospective design and the use of historical analog mammography, which prevented us from refining tiny details of the calcifications evaluated by the radiologist. However, these circumstances allowed us to track women time enough to properly assess the impact of BAC on the development of cardiovascular events. Futures studies with higher number of women and new technologies, such as digital mammograms or tomosynthesis, may contribute to improving BAC evaluation, e.g., in terms of bilaterality, number, and intensity (mild, moderate or severe). On the other hand, to avoid depending only on radiologists and their eye-observation, we need new methods of BAC quantification in order to perform a better radiological identification [27]. In this way, we could predict exactly the risk of cardiovascular disease and define better individualized preventive actions [27]. Along the same lines, AlGhamdi et al. [28] recently presented a model of deep neural network (deep learning) capable of accurately detecting BAC in mammograms, which can be used to automatically mark calcifications on the original image. The results exhibit a more accurate assessment of BAC than expert radiologists, with fewer mistakes.

Our results point to the importance of BAC on mammograms to identify women with increased cardiovascular risk and death. This may bring us the opportunity to improve preventive cardiovascular measures.

## Figures and Tables

**Table 1:** Clinical- pathological characteristics of the 128 patients in each group at the beginning and the end of the follow-up period.

	At the Beginning			At the End of the Follow-Up Period		
	Women with BAC (n = 128)	Women without BAC (n = 128)	*p* Value *	Women with BAC (n = 128)	Women without BAC (n = 128)	*p* Value *
Hypertension	38 (29.7%)	31 (24.2%)	0.398	101 (78.9%)	86 (67.2%)	0.049
Diabetes mellitus	9 (7%)	4 (3.1%)	0.255	36 (28.6%)	35 (27.3%)	0.938
Dyslipidemia	23 (18%)	34 (26.6%)	0.133	81 (63.3%)	81 (63.3%)	1.000
Obesity	35 (27.3%)	22 (17.2%)	0.071	47 (36.8%)	46 (35.9%)	1.000
Tobacco	3 (2.3%)	7 (5.5%)	0.333	n.a	n.a	-

BAC, breast arterial calcifications; Data are reported as number of cases (%) * Chi-squared test. n.a: not available.

**Table 2:** Relative risk of the occurrence of cardiovascular events during the follow-up period in women with or without BAC.

	Women with BAC (n = 128)		Women without BAC (n = 128)	
Cardiovascular Events	Nº (%) of Events (*)	RR (95% CI)	Nº (%) of Events (*)	RR (95% CI)
Ischemic heart disease	26 (20.3)	3.25 (1.53–6.90)	8 (6.3)	0.85 (0.77–0.94)
Hypertensive heart disease	35 (27.3)	2.85 (1.59–5.09)	16 (12.5)	0.79 (0.69–0.89)
Valvular heart disease	37 (28.9)	2.19 (1.28–3.75)	13 (10.2)	0.83 (0.73–0.94)
Congestive heart failure	33 (25.8)	2.06 (1.19–3.56)	16 (12.5)	0.85 (0.75–0.96)
Atrial fibrillation	33 (25.8)	1.83 (1.09–3.08)	18 (14.1)	0.86 (0.76–0.98)
Peripheral vascular disease	28 (21.9)	2.8 (1.42–5.52)	10 (7.8)	0.85 (0.76–0.94)
Ischemic stroke	9 (7)	1.5 (0.55–4.09)	6 (4.7)	0.98 (0.92–1.04)
Hemorrhagic stroke	2 (1.6)	1.0 (0.14–6.99)	2 (1.6)	1.0 (0.97–1.03)
Lacunar infarction	29 (22.7)	2.23 (1.21–4.09)	13 (10.2)	0.86 (0.77–0.96)
Total events	232	1.66 (1.31–2.1)	102	0.53 (0.39–0.72)

BAC, Breast arterial calcification; Nº: number of events; RR, relative risk; CI; confidence interval. (*) Note that a woman may have had more than one event during the follow-up period.

**Table 3:** Multivariate analysis of the relationship of classical risk factors at the beginning of the study and BAC with cardiovascular event-free and cardiovascular event-specific survival.

		Cardiovascular Event-Free Survival		Cardiovascular Event-Specific Survival	
Risk Factors	Nº of Women	Event Frecuency	HR (95% CI)	Event Frecuency	HR (95% CI)
BAC/no BAC	128/128	88/53	1.9 (1.3–2.7) ***	16/1	14.1 (1.9–107.6) **
Hypertension/no Hypertension	69/187	38/103	-	11/6	6.6 (2.4–18.4) ***
DL/no DL	57/199	30/111	-	2/15	-
DM/no DM	13/243	12/129	2.6 (1.4–4.8) **	3/14	4.9 (1.4–17.9) *
Obesity/no Obesity	57/199	33/108	-	4/13	-
Tobacco/no tobacco	10/246	7/134	-	0/17	-

BAC, breast arterial calcifications; DL, dyslipidemia; DM, diabetes mellitus; HR, hazard ratio; CI, confidence interval. Only the first event was considered for every woman. * *p* < 0.05; ** *p* < 0.005; *** *p* < 0.0001.

## Data Availability

Data available on request.

## Abbreviations

A.M., Arancha Martín; APE, Acute pulmonary edema; APTE, Acute pulmonary thromboembolism; BAC, Breast arterial calcification; BMI, Body mass index; C.d.B.M., Covadonga del Blanco Martínez; COVID-19, Coronavirus Disease-2019; CRA, Cardiorespiratory arrest; CVRF, Cardiovascular risk factors; DL, Dyslipidemia; DM, Diabetes Mellitus; HBP, High blood pressure; IHD, Ischemic heart disease; LDL-C, Low-density lipoprotein cholesterol; N.E., Noemi Eiro; N.G.-G., Natalia González-Galiano; O.F.-G., Oscar Fernández-Guinea; PVD, Peripheral vascular disease; SPSS, Statistical Package for the Social Sciences.

## References

[1] Kemmeren J.M., van Noord P.A., Beijerinck D., Fracheboud J., Banga J.D., van der Graaf Y. (1998). Arterial calcification found on breast cancer screening mammograms and cardiovascular mortality in women: The DOM Project. Doorlopend Onderzoek Morbiditeit en Mortaliteit. Am. J. Epidemiol..

[2] Wallin R., Wajih N., Greenwood G.T., Sane D.C. (2001). Arterial calcification: A review of mechanisms, animal models, and the prospects for therapy. Med. Res. Rev..

[3] Wang Q., Jin L., Wang H., Tai S., Liu H., Zhang D. (2018). AWRK6, A Synthetic Cationic Peptide Derived from Antimicrobial Peptide Dybowskin-2CDYa, Inhibits Lipopolysaccharide-Induced Inflammatory Response. Int. J. Mol. Sci..

[4] Abou-Hassan N., Tantisattamo E., D’Orsi E.T., O’Neill W.C. (2015). The clinical significance of medial arterial calcification in end-stage renal disease in women. Kidney Int..

[5] Ahn K.J., Kim Y.J., Cho H.J., Yim H.W., Kang B.J., Kim S.H., Kim H.S., Kim K.T., Lee J.H., Whang I.Y. (2011). Correlation between breast arterial calcification detected on mammography and cerebral artery disease. Arch. Gynecol. Obstet..

[6] Jiang X., Clark M., Singh R.K., Juhn A., Schnatz P.F. (2015). Association of breast arterial calcification with stroke and angiographically proven coronary artery disease: A meta-analysis. Menopause.

[7] Chadashvili T., Litmanovich D., Hall F., Slanetz P.J. (2016). Do breast arterial calcifications on mammography predict elevated risk of coronary artery disease?. Eur. J. Radiol..

[8] Soylu A., Soylu K., Aydın R., Uzunkaya F., Aslan K., Polat A.V. (2019). Calcification of breast artery as detected by mammography: Association with coronary and aortic calcification. Turk. J. Med. Sci..

[9] McLenachan S., Camilleri F., Smith M., Newby D.E., Williams M.C. (2019). Breast arterial calcification on mammography and risk of coronary artery disease: A SCOT-HEART sub-study. Clin. Radiol..

[10] Fathala A.L., Alabdulkarim F.M., Shoukri M., Alanazi M. (2018). Association between breast arterial calcifications found on mammography and coronary artery calcifications in asymptomatic Saudi women. Ann. Saudi. Med..

[11] van Noord P.A., Beijerinck D., Kemmeren J.M., van der Graaf Y. (1996). Mammograms may convey more than breast cancer risk: Breast arterial calcification and arterio-sclerotic related diseases in women of the DOM cohort. Eur. J. Cancer Prev..

[12] Kemmeren J.M., Beijerinck D., van Noord P.A., Banga J.D., Deurenberg J.J., Pameijer F.A., van der Graaf Y. (1996). Breast arterial calcifications: Association with diabetes mellitus and cardiovascular mortality. Work in progress. Radiology.

[13] Moshyedi A.C., Puthawala A.H., Kurland R.J., O’Leary D.H. (1995). Breast arterial calcification: Association with coronary artery disease. Work in progress. Radiology.

[14] de Waard F., Collette H.J., Rombach J.J., Baanders-van Halewijn E.A., Honing C. (1984). The DOM project for the early detection of breast cancer, Utrecht, The Netherlands. J. Chronic. Dis..

[15] Gennarelli M., Jedynak A., Forman L., Wold E., Newman R.B., Dhand A., Kapoor A., Jafri F., Pal S., Pandav J. (2021). The potential impact of mammographic breast arterial calcification on physician practices in a primary care setting. Future Cardiol..

[16] Iribarren C., Chandra M., Lee C., Sanchez G., Sam D.L., Azamian F.F., Cho H.M., Ding H., Wong N.D., Molloi S. (2022). Breast Arterial Calcification: A Novel Cardiovascular Risk Enhancer Among Postmenopausal Women. Circ. Cardiovasc. Imaging.

[17] Pidal D., Sánchez Vidal M.T., Rodríguez J.C., Corte M.D., Pravia P., Guinea O., Pidal I., Bongera M., Escribano D., González L.O. (2009). Relationship between arterial vascular calcifications seen on screening mammograms and biochemical markers of endothelial injury. Eur. J. Radiol..

[18] Saá J., Fernández-Guinea O., García-Pravia P., Fernandez-Garcia B., Eiró N., del Casar J.M., Venta R., Baamonde B., Vizoso F.J. (2014). Relationship between breast arterial calcifications seen on screening mammograms and age-related macular degeneration. Acta Ophthalmol..

[19] Brown A.L., Wahab R.A., Zhang B., Smetherman D.H., Mahoney M.C. (2022). Reporting and Perceptions of Breast Arterial Calcification on Mammography: A Survey of ACR Radiologists. Acad Radiol.

[20] Chen L.Y., Sotoodehnia N., Buzkova P., Lopez F.L., Yee L.M., Heckbert S.R., Prineas R., Soliman E.Z., Adabag S., Konety S. (2013). Atrial fibrillation and the risk of sudden cardiac death: The atherosclerosis risk in communities study and cardiovascular health study. JAMA Intern. Med..

[21] Coronado B.E., Griffith J.L., Beshansky J.R., Selker H.P. (1997). Hospital mortality in women and men with acute cardiac ischemia: A prospective multicenter study. J. Am. Coll. Cardiol..

[22] Margolies L., Salvatore M., Hecht H.S., Kotkin S., Yip R., Baber U., Bishay V., Narula J., Yankelevitz D., Henschke C. (2016). Digital Mammography and Screening for Coronary Artery Disease. JACC Cardiovasc. Imaging.

[23] Ružičić D., Dobrić M., Vuković M., Hrnčić D., Đorđević S., Ružičić M., Aleksandrić S., Đorđević-Dikić A., Beleslin B. (2018). The correlation of SYNTAX score by coronary angiography with breast arterial calcification by digital mammography. Clin. Radiol..

[24] Yoon Y.E., Kim K.M., Han J.S., Kang S.H., Chun E.J., Ahn S., Kim S.M., Choi S.I., Yun B., Suh J.W. (2019). Prediction of Subclinical Coronary Artery Disease With Breast Arterial Calcification and Low Bone Mass in Asymptomatic Women: Registry for the Women Health Cohort for the BBC Study. JACC Cardiovasc. Imaging.

[25] Disthabanchong S., Boongird S. (2017). Role of different imaging modalities of vascular calcification in predicting outcomes in chronic kidney disease. World J. Nephrol..

[26] Gernaat S.A.M., van Velzen S.G.M., Koh V., Emaus M.J., Išgum I., Lessmann N., Moes S., Jacobson A., Tan P.W., Grobbee D.E. (2018). Automatic quantification of calcifications in the coronary arteries and thoracic aorta on radiotherapy planning CT scans of Western and Asian breast cancer patients. Radiother. Oncol..

[27] Trimboli R.M., Codari M., Guazzi M., Sardanelli F. (2019). Screening mammography beyond breast cancer: Breast arterial calcifications as a sex-specific biomarker of cardiovascular risk. Eur. J. Radiol..

[28] AlGhamdi M., Abdel-Mottaleb M., Collado-Mesa F. (2020). DU-Net: Convolutional Network for the Detection of Arterial Calcifications in Mammograms. IEEE Trans. Med. Imaging.

